# Biochars from Cotton Seed, Camelia Seed Shell, and Coffee Ground in Modification of Asphalt: Fundamental Properties, Rheological Performance, and Inhibition of VOC Emissions

**DOI:** 10.3390/ma18071504

**Published:** 2025-03-27

**Authors:** Xiao Zhang, Yi Zhou, Yongjie Xue

**Affiliations:** State Key Laboratory of Silicate Materials for Architectures, Wuhan University of Technology, Wuhan 430070, China; fans-cooler@outlook.com (X.Z.); joejoe-98@live.com (Y.Z.)

**Keywords:** asphalt modification, biochar, rheology

## Abstract

With the increasing requirement for asphalt modification, a new environmentally friendly asphalt modifier is needed. In this study, three varieties of biomass, cotton seed (CO), camelia seed shell (CA), and coffee ground (CG), were chosen for biochar preparation and asphalt modification to find an environmentally friendly asphalt modifier. A segregation test was applied to evaluate the storage stability of the modified asphalt. A dynamic shear rheometer (DSR) temperature sweep and frequency sweep were used to characterize the high-temperature performance. The low-temperature performance was evaluated by the bending beam rheometer (BBR) test. The DSR results indicate that the rutting factor increase for modified asphalt at high temperatures is CO ≈ CG > CA, and a high temperature could reflect the biochar’s properties better in modified asphalt. Furthermore, the low-temperature deterioration is well controlled in CO and CA biochar-modified asphalt. Finally, the volatile organic compound (VOC) emission behavior was evaluated using gas chromatography–mass spectrometry (GC-MS).

## 1. Introduction

Asphalt, a byproduct of the petroleum industry, is an organic viscoelastic material with excellent physical properties. Asphalt binder is widely used in the construction of arterial highways and urban roads for its advantages, including low passing noise, low vibration, comfortable driving experience, and simple maintenance. However, asphalt binder faces various challenges, such as heavy loads and extreme climates, posing difficult challenges for virgin asphalt. Adding a modifier to asphalt binders and preparing a modified asphalt binder are effective solutions to these problems. Currently, styrene–butadiene–styrene block copolymer (SBS), styrene–butadiene rubber (SBR), polyethylene (PE), etc., [1,2,3,4,5,6,7], are the most conventional modifiers considering both economy and performance. SBS could improve the high-temperature performance and reduce the temperature sensitivity of asphalt to enhance the anti-deformation ability of asphalt binders [1,2,3,8]. SBR is the main material of tires; thus, recycled tires are fantastic feedstock for SBR asphalt modifiers. A proper content of SBR in asphalt is an obvious benefit for ductility at low temperatures, elastic recovery rate, and viscosity [4,9,10].

The production of asphalt and modifiers as well as storage is a crucial part of the life cycle of asphalt pavements [11]. As China aims to hit peak carbon dioxide emissions in 2030 and achieve carbon neutrality in 2060, the energy consumption and carbon emissions from the production of pavement materials account for a significant portion of the carbon emissions life cycle assessment of highway facilities. Not only does the production of modifiers consume energy, but the energy required to produce the raw materials also contributes substantially. For rubber asphalt, the value is 42,700 MJ/t and 34,900 MJ/t, respectively. And the comprehensive energy consumption of plastic rubber-modified asphalt and SBS-modified asphalt mixture is 678.8 MJ/t and 743.8 MJ/t, within which the consumption for modifiers is 36 MJ and 98.8 MJ, respectively [12]. These substantial energy consumptions indirectly lead to considerable carbon emissions. In response to carbon reduction and the construction of a circular economy, new low-carbon asphalt modifiers are urgently needed.

Biochar, a product of biomass pyrolysis, emerges as a promising low-carbon modifier alternative for asphalt modification [13]. The energy consumption of biochar production largely depends on the pyrolysis temperature, a parameter that affects both pyrolysis time and yield. However, the overall energy and carbon emissions are still lower than those of the traditional modifiers mentioned above. The carbon in biochar primarily originates from lignin [14], hemicellulose, and cellulose [15] in biomass, which means biomass with high contents of these components is suitable for biochar production. The requirements for feedstock for biomass pyrolysis are not strict, as it can utilize agricultural waste [16,17] without dedicated cultivation requirements, providing a sustainable way to handle agricultural wastes.

The porous structure and surface functional groups of biochar allow it to interact with asphalt, thus enhancing asphalt’s performance. Kumar et al. [17] investigated biochar-modified asphalt with biochar contents ranging from 0% to 20% and found that biochar significantly increases asphalt’s viscosity and rutting resistance. However, a high biochar content deteriorates fatigue and aging resistance. Zhang et al. [18] studied the influence of biochar particle size on asphalt performance, revealing that smaller biochar particles outperformed larger ones. Furthermore, compared with graphene, the surface of biochar is much tougher, with an abundant porous structure. Ma et al. [19] pointed out that the degradation in the low-temperature performance of biochar-modified asphalt can be managed by adjusting the biochar dosage. Hu et al. [20] prepared corn stalk biochar through the hydrothermal method and explored the molecular weight distribution of biochar-modified asphalt and its relation to biochar contents, finding that a 6 wt% dosage allows for uniform distribution in asphalt with minimal clustering. Zhao et al. [21] demonstrated that biochar enhances the moisture resistance of hot-mixed biochar-modified asphalt mixtures, with a lower impact on crack resistance compared to carbon black and carbon fiber.

Except for modification, biochar also removes the volatile organic compounds (VOCs) in asphalt, which are hazardous and harmful to the environment and human health. Zhou et al. [22] find that biochar could improve the adsorption energy of asphalt, and the adsorption energy would increase with temperatures. Li et al. [23] simulated three representative scenarios with various VOC emission concentrations. Alkenes (n < 4), alkanes (n > 6), aldehydes, and alkylbenzenes were the main ingredients for the asphalt VOC emission. Zhou et al. [24] compared differential scanning calorimetry (DSC) curves of virgin and biochar-modified asphalt, indicating the chemical adsorption mechanism in VOC inhibition.

While the existing studies on biochar-modified asphalt primarily focus on one single biochar or biomass feedstock, few studies compare the effects of multiple biochar types. The problem of whether different biochars have distinct effects on asphalt performance remains unresolved. Moreover, the relationship between biochar properties and modified asphalt performance is not well understood. Considering both economic and environmental factors, we selected coffee grounds, camellia seed shells, and cotton seed, three types of agricultural and agroindustry wastes, to prepare biochar asphalt modifiers. As coffee is the most popular beverage, the use of its residue, coffee grounds, holds promise. Cotton seed is a byproduct of the cotton industry, and camellia seed shells are the shells of a commonly grown oilseed in the Yangtze River basin. These biomasses, rich in lignin, cellulose and hemicellulose [15,25,26,27], are ideal raw materials for biochar production. This work aims to find the best biochar asphalt modifier among the 3 types of biochar and investigate the performance improvement of biochar-modified asphalt. We prepared biochar from these three biomasses, performed basic characterizations, and prepared biochar-modified asphalt with dosages of 1%, 6%, and 9% by the weight of virgin asphalt. Penetration, softening point, and ductility tests were conducted to determine the optimal biochar content. The modified asphalt with selected content was further evaluated through DSR and BBR tests, comparing the performance of three types of biochar-modified asphalts. This study provides insights into how different biochars affect asphalt performance, potentially contributing to agricultural waste management and green economy construction. In the end, the biochar-modified asphalt with the best comprehensive physical properties was detected to evaluate the VOC emission behavior through thermal desorption–gas chromatography–mass spectrometry (TD-GC-MS).

## 2. Materials and Methods

### 2.1. Materials

Three varieties of biomass: cotton seed (CO), camelia seed shell (CA) and coffee ground (CG), were selected as biochar feedstocks, among which CO and CA were collected from local farms while CG was bought from Starbucks (Wuhan, Hubei Province, China). Asphalt 70# was applied in the preparation of modified asphalt binder, with related parameters presented in Table 1.

### 2.2. Asphalt Modification

The biomass was first dried in an oven at 80 °C for 12 h. Biochar was then produced via slow pyrolysis, heating the biomass to 600 °C at a rate of 10 °C/min, with a residence time of 90 min under a nitrogen atmosphere at 80 mL/min. Then, the produced biochar was ground with a planet grinding machine for 40 min, as the particle size may influence the biochar dispersion and modification effect. No further treatment was applied. After grinding, the biochar powder was blended and agitated with virgin asphalt using a high-speed shearing mixer for 40 min. Biochar content was set at 1%, 6%, and 9% by the weight of virgin asphalt, with a shearing rate of 3000 rpm at 135 °C [31]; the asphalt was heated with an oil bath.

### 2.3. Characterization of Biochar

#### 2.3.1. Elemental Analysis

Elemental analysis for the 3 varieties of biochar was conducted, respectively, in order to measure the contents of carbon, hydrogen (H), oxygen (O), nitrogen (N) and sulfur (S). The C, H, N and S contents were measured by a Vario EL Cube elementar (Elementar, Hanau, Germany), while O was measured using a Vario EL Marco Cube elementar (Elementar, Hanau, Germany).

#### 2.3.2. BET

The Brunauer–Emmett–Teller (BET) specified surface area and pore size distribution of the biochar samples were measured using a Micromeritics ASAP 2420 Surface Area & Porosimetry System (Micromeritics, Norcross, GA, USA) with nitrogen adsorbent at a temperature of 77 K.

#### 2.3.3. FTIR

A Fourier transform infrared (FTIR) spectrometer (Bruker Alpha, Rheinstetten, Germany) was utilized to obtain the infrared absorption spectra of the biochar samples from the wave number of 4000 cm^−1^–400 cm^−1^ with 2 cm^−1^ resolution, employing the potassium bromide (KBr) pellet method for biochar sample preparation.

### 2.4. Evaluation of Biochar-Modified Asphalt Properties

#### 2.4.1. Conventional Properties

The penetration, softening point and ductility tests were conducted according to ASTM D5, D36 and ASTM D113-17, respectively. The penetration test was conducted at 15 °C, 25 °C and 30 °C, by which the penetration indexes were evaluated using Equations (1) and (2) to evaluate the temperature sensitivity of biochar-modified asphalt.

(1) $$lgP=K+A_{lgPen}T$$

(2) $$PI=\frac{20-500A_{lgPen}}{1+50A_{lgPen}}$$

#### 2.4.2. Segregation Test

For modified asphalt, the storage stability is critical in the evaluation of long-term properties [4,32,33,34], which is considered through the compatibility of asphalt with modifiers. A delayed segregation test was applied to evaluate the storage stability of biochar-modified asphalt and the effect of biochar variety. The test was conducted according to ASTM D7173-14 [35] with a 48 h soaking, and ASTM D36 was applied to measure the softening point difference.

#### 2.4.3. Temperature Sweep

In order to assess the rheological properties of biochar-modified asphalt across medium to high temperature, dynamic shear tests were carried out in this research. Temperature sweep and frequency sweep tests were conducted on the virgin asphalt and 3 varieties of modified asphalt in 12% strain-controlled mode using an Anton Paar (Graz, Austria) SmartPave 102e rheometer. A PP25 plate was used in this test, where the sample thickness was 1 mm.

Temperature sweep is a common method to evaluate the temperature sensitivity and high-temperature performance of asphalt binders. The test temperature was from 30 °C to 80 °C with a loading frequency of 10 rad/s; 3 data points were equidistantly taken every 5 °C at the test temperature range. Through the test, basic rheological properties, complex shear modulus G* and phase angel *δ* were obtained, from which the rutting factor G*/sin δ and critical temperature were calculated.

#### 2.4.4. Frequency Sweep

The modulus of typical amorphous polymers increases with loading frequency but decreases when the temperature increases. And curves of the instantaneous modulus as a function of time do not change their shape as the temperature changes, only shifting left or right. It could determine temperature-dependent mechanical properties of linear viscoelastic materials from known properties at a reference temperature. It offers a method to predict curves at various frequencies and temperatures using a shift factor based on the master curve in a given test condition. And this rule is called the time–temperature superposition (TTS) principle.

The TTS principle avoids the inefficiency of measuring polymer behavior over long periods of time, which usually costs a lot of time. According to the introduction above, frequency sweep tests were conducted from 0.01 Hz to 10 Hz at temperatures between 30 °C and 60 °C with an increment of 10 °C. Serval methods were applied to characterize the viscoelastic behavior of biochar-modified asphalt.

The master curve is an effective method in the research of asphalt TTS behavior. It shows the dependance of G* and δ for loading frequency. As a popular method in the research of polymer TTS behaviors, it is also effective for deducing the change of microscopic structure in modified asphalt. For asphalt materials, the master curve could be constructed by the Williams–Landel–Ferry (WLF) model, as shown in Equation (3).

(3) $$\log\alpha_{T}=-\frac{C_{1}\left(T-T_{0}\right)}{C_{2}+\left(T-T_{0}\right)}$$

where *C*_1_ and *C*_2_ are positive constants depending on the material and reference temperature; *α_T_* refers to the shift factor and *T*_0_ is reference temperature. Therefore, through the TTS principle, we can obtain complex dynamic moduli like asphalt complex shear modulus at a required frequency using Equations (4) and (5).

(4) $$G^{\prime }\left(\omega ,T\right)=G^{\prime }\left(\alpha_{T}\omega ,T_{0}\right)$$

(5) $$G^{\prime \prime }\left(\omega ,T\right)=G^{\prime \prime }\left(\alpha_{T}\omega ,T_{0}\right)$$

Han et al. [36,37] discovered that the order–disorder transition of block copolymers could be presented by the double logarithmic diagram of *G′* versus *G″*. Though it does not show the relationship between modulus and frequency directly, the higher the modulus, the lower the frequency. Therefore, this method is now widely applied in the research of asphalt. In the Han curve, the graph of *G′* and *G″* is linear-like, following Equation (6) [37].

(6) $${logG}^{\prime }=\mathrm{Alog}G^{\prime \prime }+B$$

where *A* and *B* are constants, in relation with the density, experimental temperature and molecular weight. In the low-frequency terminal zone of the curve, according to previous investigations [38], the plots are expected to have a slope of 2 in the term region for all homogeneous polymeric liquids. This phenomenon is an embodiment of the micro phase change of asphalt at low frequency at the macro scale.

#### 2.4.5. Bending Beam Rheometer Test

Cracking is a common problem for asphalt pavement at low temperature. Since the aggregate properties would not be affected by temperature, the performance of the asphalt binder plays a critical role in cracking resistance. The cracking resistance at low temperature of biochar-modified asphalt was evaluated by bending beam rheometer (BBR) test at −12 °C, −18 °C and −24 °C according to ASTM D6648-16 [39]. The heated modified asphalt was poured into a mold and shaped into a beam specimen (127 mm × 6.35 mm × 12.7 mm). Then, the flexural creep stiffness *S(t)* and creep rate (*m* value) were measured at each test temperature. According to Superpave, the *S(t)* of asphalt binder should be less than 300 MPa, while the *m* value must be more than 0.3 [40].

### 2.5. Asphalt VOC Emission Evaluation

VOCs emitted by the samples were collected by an adsorption tube consisting of the Combination 1 adsorption tube containing Tenax GR and Carbopack B, as stipulated in HJ 734-2014 [41]. Then, the VOCs were detected by a TD-GC-MS (TD: PerkinElmer (Waltham, MA, USA) Turbo matrix ATD 350, GC-MS: Agilent Atomx P&T-Agilent 7890B-5977B, Santa Clara, CA, USA). Qualitative analysis was conducted through NIST mass spectroscopy library. Gas chromatography was applied to record the chromatograms of modified asphalt. Through peak searching, the area of the peaks is calculated and normalized. Considering the complexity and diversity, some original and representative components in asphalt VOCs were detected as the references whose emissions in virgin and modified asphalt are compared, respectively, through which the VOC inhibition of the biochar modifier is evaluated.

## 3. Results and Discussion

### 3.1. Biochar Characterization

**Figure 1:**
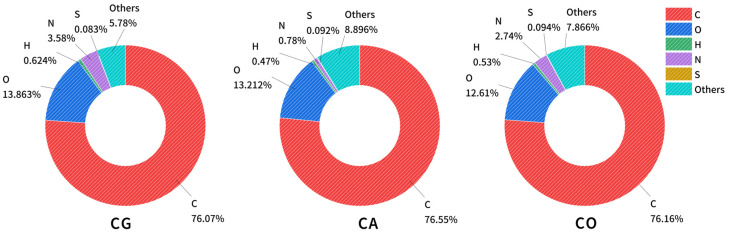
The element contents for biochar.

The porosity and surface reactivity of biochar surpass traditional carbon-based fillers. The elemental analysis (Figure 1) indicates carbon contents reaching approximately 76% for all three biochars, confirming complete pyrolysis of lignin, cellulose, and hemicellulose. Differences in H, O, and N content were observed. The H content was highest in CG (0.624%), followed by CO (0.53%) and CA (0.47%). CG has the highest oxygen content at 13.86%, followed closely by CA at 13.21% and CO at 12.61%. Notably, CG contains 3.58% nitrogen, while CO has 2.74%, with CA showing nearly no nitrogen content.

**Figure 2:**
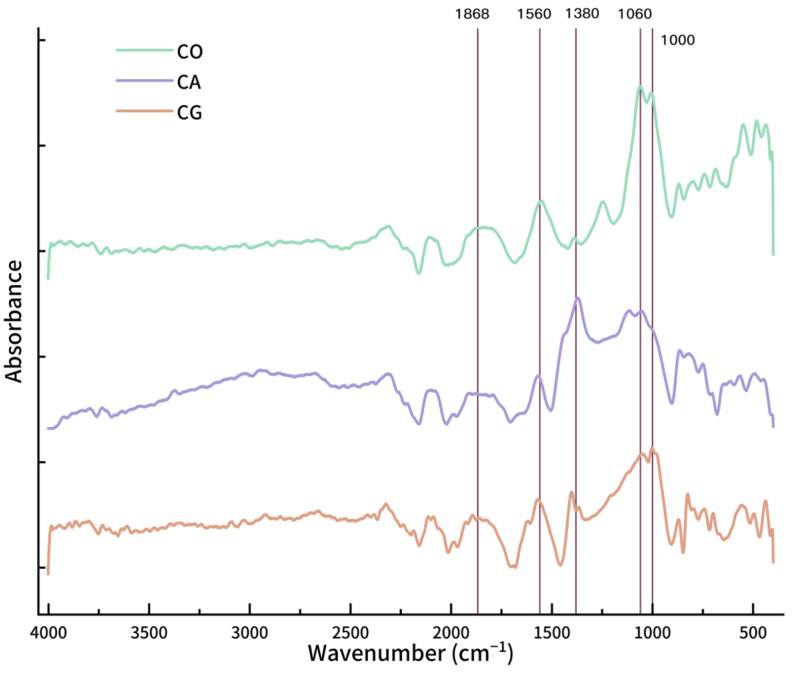
Infrared adsorption spectra for biochar.

The surface functional groups could influence physical and chemical properties of biochar [42,43]. FTIR spectra (Figure 2) show the surface functional groups for the three biochars. Peaks between 1868 and 1560 cm^−1^ correspond to the stretching vibrations of C=O, C=C, and potentially some C=N [44] and N-O bonds, with CO and CG displaying a stronger absorption than CA. The absorption peak at 1380 cm^−1^, mainly from aromatic rings and C-H bonds in methyl and methylene groups [45], is stronger in CA and CG, but weaker in CO. The double peaks around 1100–1000 cm^−1^ indicate C-O and C-N [46] stretching vibrations.

Analyzing the spectra from 4000 to 2400 cm^−1^, combined with the elemental contents, it is obvious that CG reaches the highest absorptivity at this range. It is speculated that a considerable part of it is brought by N-H [47]. The double peaks at 1060 cm^−1^ for CO are noticeably the strongest, and there should also be more C-N bonds in addition to C-O bonds.

**Figure 3:**
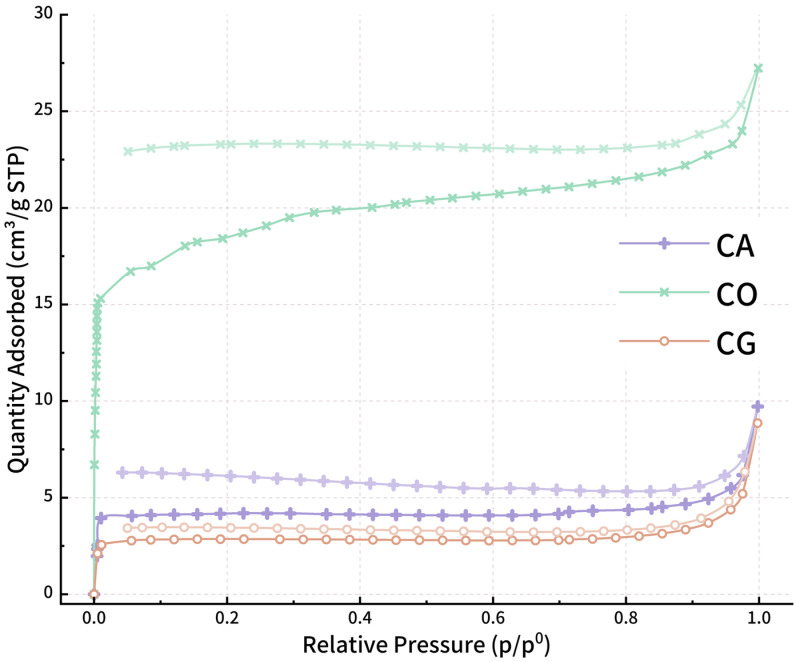
Isotherm adsorption and desorption plot.

Both surface area and porosity are crucial physical properties for biochar [48,49]. Table 2 lists the specific surface area and pore volume for the 3 varieties of biochar through BET, BJH and t-Plot methods. It reveals that CO has the highest specific surface area of 66.42 m^2^/g and pore volume of 0.04 cm^3^/g among the biochar. Figure 3 shows the isothermal adsorption/desorption plots of the three varieties of biochar. The line within higher saturation accounts for the adsorption process and the other for the desorption process. The isotherms do not close, indicating adsorption hysteresis [50], characteristic of a type Ⅳ isotherm [51] with an H2 hysteresis loop [52,53]. In the low relative pressure region, the adsorption of CO increases rapidly and saturates after reaching a certain relative pressure as the adsorption rate slows down. It highlights a relatively high micropore content, consistent with the high specific surface area from the t-Plot method. And then the shape of the 3 adsorption isotherms tends to be consistent, indicating a comparable mesoporous and microporous distribution among the 3 varieties of biochar.

### 3.2. Performance of Modified Asphalt

#### 3.2.1. Selection of Biochar Content Based on Conventional Properties

Table 3 presents the basic properties of biochar-modified asphalt at different biochar contents (1%, 6%, and 9%). The penetration of 9 varieties of modified asphalt were measured at 15 °C, 25 °C, and 30 °C and penetration indexes were calculated. It shows a decrease in penetration values with increasing biochar content, with biochar’s modification effect on asphalt increasing with higher test temperatures. It is obvious that the penetration of modified asphalt decreases with the contents of biochar, which is also in line with the discovery from other related studies about biochar-modified asphalt. Furthermore, biochar modification could lower the penetration of asphalt; for virgin asphalt, this value is −0.33. While the modification effect for 1% is negligible, when biochar content reaches 6%, the penetration index is −0.80, which is much lower than virgin asphalt. Biochar-modified asphalt’s softening point also rises with biochar content, though the increase was modest. The modified asphalt with the highest softening point is CA-9%, only 2.64 °C ahead of virgin asphalt. However, excessive biochar content (9%) reduces low-temperature ductility significantly, dropping below the threshold of 20 cm at 15 °C, which is unsuitable for engineering applications [54]. Considering both high- and low-temperature performance, a 6% biochar content was selected for further evaluations. The fluorescence microscopic image for modified asphalt at 6% content can be found in the Supplementary Materials.

#### 3.2.2. Storage Stability

**Figure 4:**
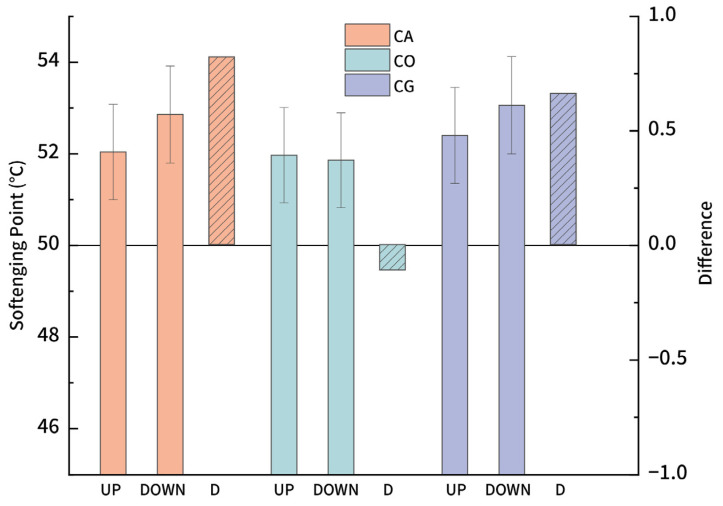
Segregation test of biochar-modified asphalt.

For asphalt modified with powdered materials, it is essential that the modifier can remain uniformly dispersed in the asphalt matrix to achieve an optimal performance. Storage stability is mainly influenced by two factors: the density of the modifier [55] and surface properties [56] of powder. At 163 °C, asphalt is in a viscous flow state; powders within mismatched densities with asphalt continuous phase may float or sink, causing segregation. Additionally, if the powder surface can interact with asphalt, it increases the resistance to segregation. Figure 4 presents the softening point differences between the upper and lower layers after a 48 h segregation test for the three biochar-modified asphalts. “D” in the figure refers to the difference of the upper and lower layers. After 48 h, the differences for 3 varieties of biochar-modified asphalt are as follows: CA 0.82 °C, CO −0.11 °C, and CG 0.66 °C, respectively, all of which are lower than ±1 °C. No phase separation was observed in the experiment. CO exhibited a smaller difference; it may result from the porosity of the biochar, which is different from others. Generally, a modifier with a difference below 2.5 °C is considered to have good compatibility, as all the modified asphalt fits the requirement, indicating excellent storage stability in this study with 6% biochar content.

#### 3.2.3. Temperature Sweep Analysis

**Figure 5:**
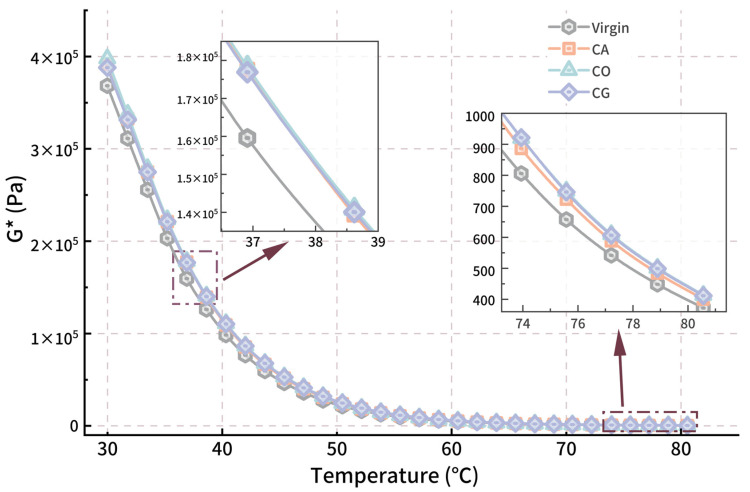
G*-T curve for modified asphalt.

**Figure 6:**
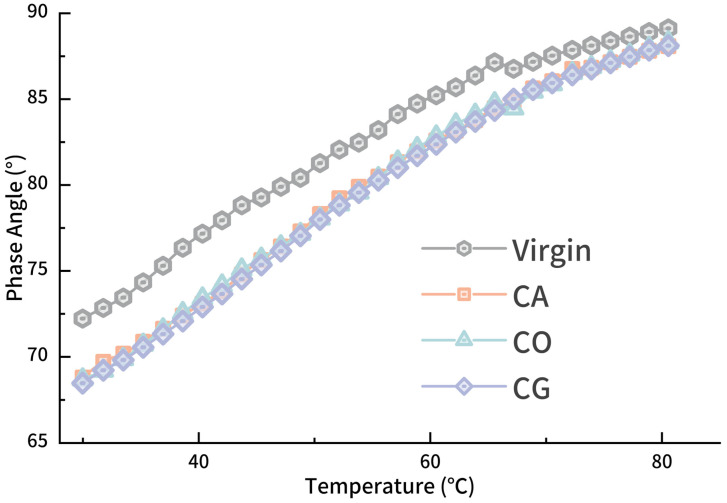
*δ-T* curve for modified asphalt.

The temperature sweep results (Figure 5 and Figure 6) show that the complex shear modulus (G*) and rutting factor G*/sin δ of the three biochar-modified asphalts decrease with increasing temperature, with the rate of decrease slowing as temperature rises. The G*-T curve fits a Boltzmann function, showing a gradual reduction in modulus as the test temperature increases, which is consistent with our expectation. Around 30 °C, the performance enhancement of modified asphalt compared to virgin asphalt is minimal, with CO-modified asphalt showing an 8% increase in modulus over virgin asphalt, while the performance differences between the biochar-modified asphalts are slight (CO > CA > CG). As the temperature rises, the enhancement for modified asphalts G* increases more significantly, and differences between the biochar-modified asphalts become more pronounced, peaking around 57 °C. At this temperature, CO- and CG-modified asphalts reach an approximate 20% increase in G*, while CA-modified asphalt shows a lower improvement (14%). At temperatures above 70 °C, asphalt begins to transition to a viscous flow state [57,58], and the modulus enhancement effect of biochar begins to decline, with a reduction to around 10% improvement at 80 °C (CO and CG). In this range, the performance of CA-modified asphalt is notably inferior to the other two groups.

The phase angle of asphalt increases linearly as temperature rises from 30 °C to 70 °C, and the difference between modified and virgin asphalt remains stable. After 70 °C, the phase angle of virgin and modified asphalts approaches the maximum of 90°, with minimal differences among the modified asphalts.

**Figure 7:**
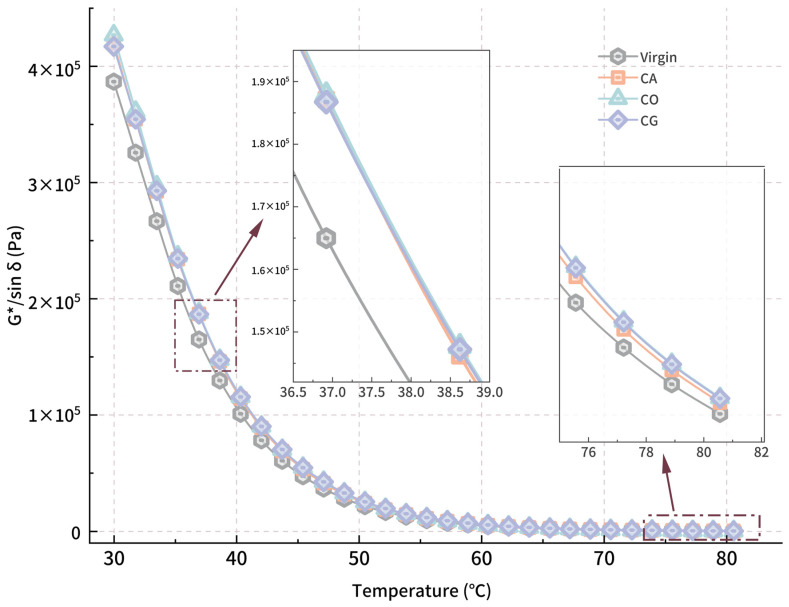
G*/sin δ-*T* curve for modified asphalt.

Since the phase angle remains above 60° throughout the test temperature range, the rutting factor (G*/sin δ) for asphalt is mainly influenced by G*. Figure 7 shows the G*/sin δ-*T* curve, with the rutting factor enhancement for each modified asphalt peaking around 57 °C (CA: 15%, CO: 19%, CG: 20%). At 60°, the *δ* for CG-modified asphalt is larger than the other 2 modified asphalts; the enhancement for CG is the most obvious. And CA accounts for the lowest enhancing effect during the whole test, while the higher the testing temperature is, the larger the difference is.

**Figure 8:**
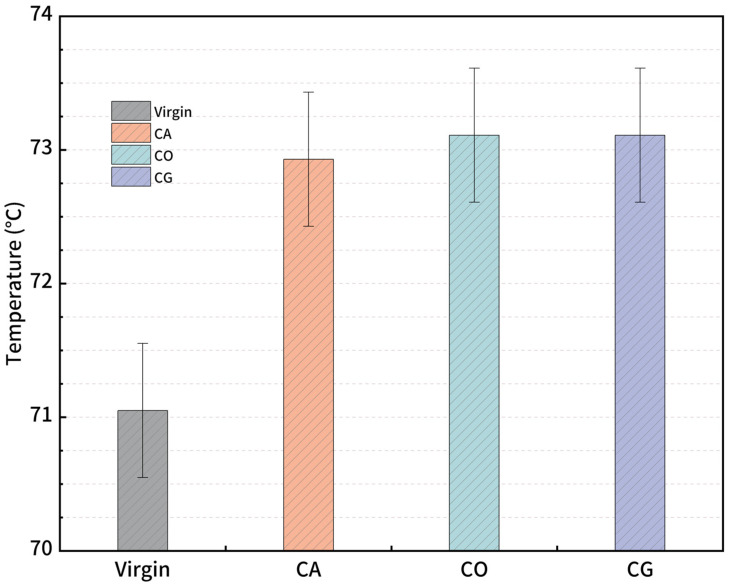
Critical temperatures of modified asphalt.

SHRP invented the critical temperature to standardize asphalt high-temperature performance evaluation, which is defined as the temperature when the rutting factor is 1 kPa. Figure 8 presents the critical temperatures for both virgin and modified asphalts, with CO- and CG-modified asphalts exceeding 73.1 °C, slightly outperforming CA-modified asphalt, at 72.9 °C. All of the 3 modified asphalt critical temperatures are higher than virgin asphalt at 72.3 °C. With the same contents, the enhancement effects for CO and CG are better than that for CA. The results suggest that the performance differences may relate to the nitrogen and associated functional groups in biochar, as CO and CG biochars contain higher nitrogen contents than CA. The differences may due to the contents and structure of the cellulose, hemicellulose and lignin in the biomass.

The biochar-modified asphalts exhibit a higher G* and lower *δ* compared to virgin asphalt, indicating a greater improvement in *G′* than in *G″*. This suggests a significant enhancement in the modified asphalt’s permanent deformation resistance and anti-rutting performance. The relatively smaller increase in *G″* may result from biochar’s adsorption of light components in asphalt [59], potentially disrupting the original colloidal structure. Among the three biochars, CO-modified asphalt shows the greatest increase in G* and the smallest reduction in *δ*, narrowing the G*/sin δ difference with other modified asphalts, likely due to the mesoporous structure.

As temperature increases, the performance difference among the three modified asphalts widens. This may be due to the fact that with the increase in testing temperature, the viscosity of asphalt decreases and the molecular motion becomes more intense, which leads to a full adsorption of light components by biochar. The adsorption capacity depends more on the property of biochar itself than on other factors, and the property difference among the 3 biochars could be fully reflected.

#### 3.2.4. Master Curve

**Figure 9:**
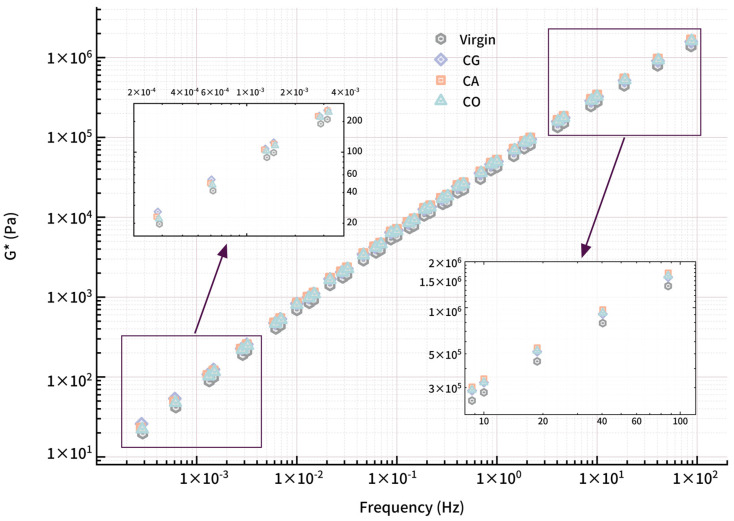
G* master curves for modified asphalt.

**Figure 10:**
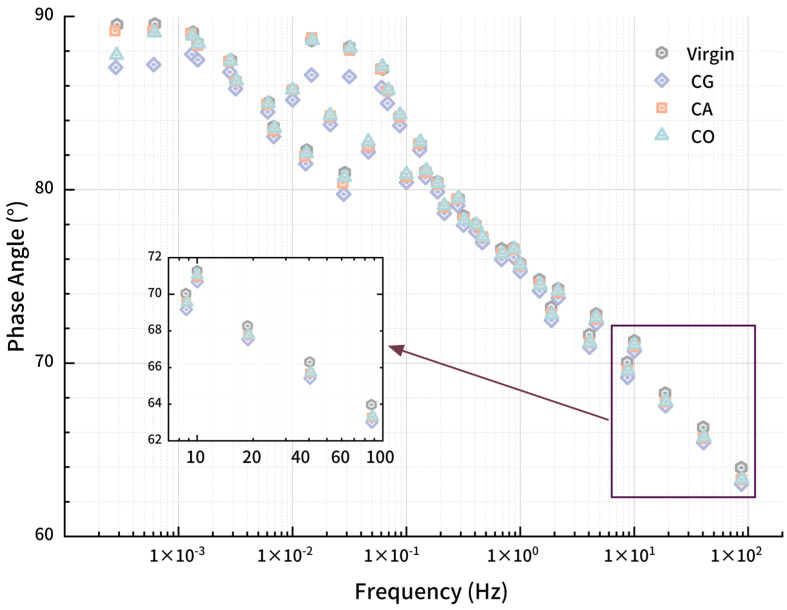
Phase angle master curves for modified asphalt.

Based on the DSR frequency sweep, master curves (Figure 9 and Figure 10) were constructed using the TTS principle at a reference temperature of 40 °C to study the rheological performance of biochar-modified asphalts over a wide frequency range. The corresponding *α_T_* values were calculated using a Python program (https://www.python.org/). Linear fitting for the G*-*f* double logarithmic curves and R^2^ shows that the modified asphalts are in a viscoelastic state where the TTS principle is validated. Master curves exhibit an increase for modified asphalts compared to virgin asphalt in complex shear modulus, especially in the high-frequency region, indicating that the modification successfully enhances the stiffness through a series of physical changes.

According to the TTS principle, the master curve in the low-frequency region represents the rheological properties for polymer materials at high temperature, while high frequency represents the properties at low temperature. In this study, the reference temperature is 40 °C; therefore, the G* master curve in the mid to low-frequency region could better reflect the rutting resistance and anti-permanent deformation ability for modified asphalt.

In the low-frequency range of the master curve, the G* of CA-modified asphalt is the highest, followed by CG, with CO exhibiting the lowest permanent deformation resistance. When *f* = 0.1 Hz, the complex shear modulus for virgin asphalt is 5784 Pa, and for modified asphalts, the values are CA 7148 Pa, CG 6874 Pa, and CO 6743 Pa. The enhancement of biochar for asphalt is evident in this region to distinguish each type of biochar; biochar properties could hardly influence the enhancement effect.

In the low-frequency region, the complex shear modulus for modified asphalts is low and increases as frequency increases, reaching the maximum at the fastest frequency, embodying the differences among biochars. In the high-frequency range, asphalt behavior approaches that of a glassy elastic material, where the complex shear modulus could reflect the asphalt’s low-temperature cracking resistance. A higher complex shear modulus at high frequency indicates a stiffer, more brittle material prone to cracking. For instance, at 87 Hz, CA-modified asphalt exhibits the highest modulus (1.68 MPa), approximately 20% higher than virgin asphalt (1.39 MPa), followed by CO (1.61 MPa) and CG (1.58 MPa).

The *δ-f* master curve shows the phase angle of modified asphalt initially increasing linearly with decreasing frequency, then the increasing rate gradually slows. The phase angle reaches a maximum at around 1 × 10^−3^ Hz before declining. CG-modified asphalt shows a phase angle curve below that of virgin asphalt, while CA and CO phase angle reduction is evident only at medium to high frequency. In the extreme low-frequency range, the phase angle approaches 90°, where *G′* is almost 0, indicating that asphalt has entered a viscous flow state, with biochar enhancing high-temperature viscosity.

#### 3.2.5. Han Curve

**Figure 11:**
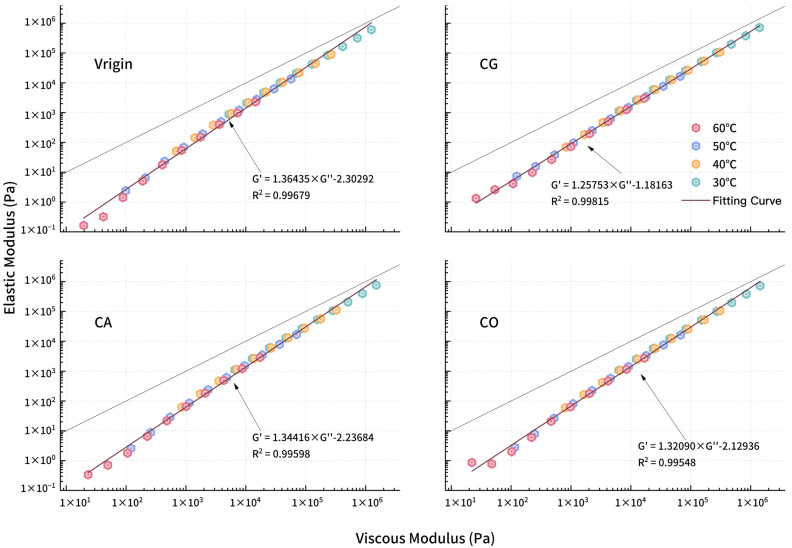
Han curves for modified asphalt.

The Han curve (Figure 11) for biochar-modified asphalt displays a linear relation between *G′* and *G″*, validating the availability of the TTS principle under test conditions. The Han curve for modified asphalt lies below the reference line (slope of 1, intercept of 0), indicating the materials are in a viscous region. Linear fitting of the Han curve shows high correlation (R^2^ > 0.99), with deviations observed only at the low-modulus, equivalent low-frequency/high-temperature end, suggesting that the modified asphalt is more elastic than virgin asphalt. Then, as frequency increases, Han curves and the corresponding fitting line gradually approach the reference line, with asphalt entering a viscoelastic state. Contrasting the Han curve for virgin and modified asphalt, the curve for virgin asphalt is at the lower left side of the modified asphalt, and both *G′* and *G″* for modified asphalts are greater than virgin asphalt, indicating the enhancement of biochar for both sides.

For the several points with the lowest modulus, *G″* of modified asphalt is much higher than *G′* while the modified asphalt is in a viscous state. At this time, biochar obviously enhances the *G″* of asphalt and the enhancement for *G′* is slight. Then, as frequency increases, the asphalts enter a viscoelastic state, with *G′* improving rapidly, embodying the enhancement.

For polymer materials, the slope of the Han curve in the low-frequency region would be larger than the entire fitting curve. The slope for homogeneous polymer materials would reach about 2 due to the phase transition at high temperature or low frequency. In Figure 10, only virgin asphalt has a visible slope change to 1.79. The slope for 3 varieties of modified asphalt deviates slightly. There are two main reasons for this phenomenon [37]. One is the test condition; the test temperature is not high enough and the loading frequency is not low enough to produce the above changes in the curve. In this study, the biochar modification treatment increases the complex shear modulus of asphalt, reduces the temperature sensitivity of modified asphalt, and makes the phase transition of asphalt difficult. The second is the polydispersity of asphalt [38]. As a mixture composed of asphaltenes, saturated components, aromatic components and colloids, asphalt has a large difference in molecular weight of different components and strong polydispersity, which can explain the phenomenon that the slope at the end region of the Han curve of virgin asphalt is less than 2. The introduction of biochar and its swelling aggravated the phenomenon of uneven molecular weight distribution in modified asphalt and inhibited the slope change of the end region of the curve.

In conclusion, biochar-modified asphalt exhibits better storage stability than SBS- and rubber-modified asphalt, while the enhancement of rheological properties is lower at high temperature.

#### 3.2.6. Low-Temperature Properties

**Figure 12:**
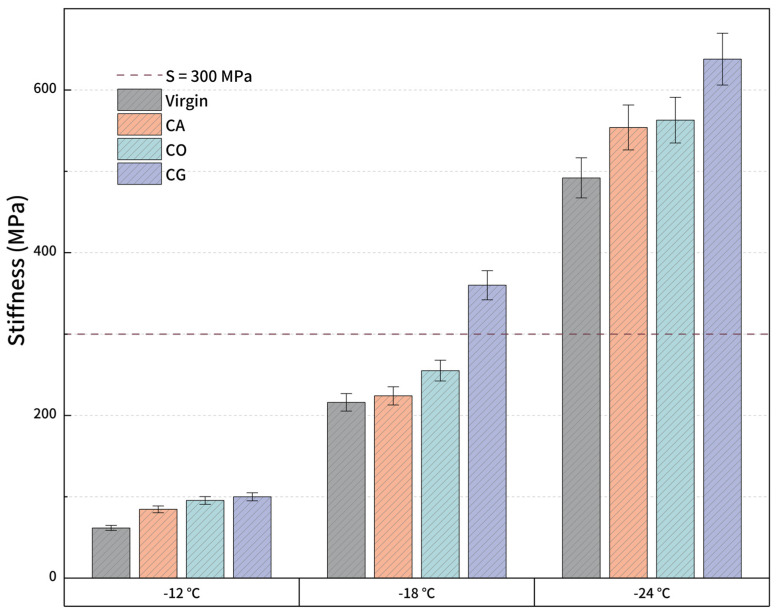
*S*(*t*) for modified asphalt.

**Figure 13:**
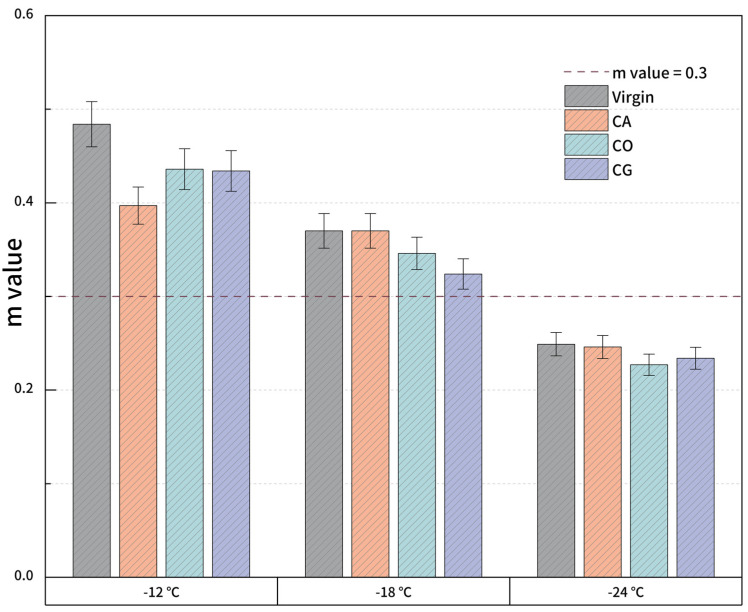
*m* value for modified asphalt.

Figure 12 and Figure 13 show the BBR results of biochar-modified asphalt, including flexural creep stiffness *S*(*t*) and *m* value. ASTM D6816 stipulates that the flexural creep stiffness of asphalt should be less than 300 MPa and the *m* value should be greater than 0.3; otherwise, the material is considered to fail. It can be discovered that the virgin asphalt and modified asphalt pass the test at −12 °C. However, the flexural creep stiffness of CG-modified asphalt at −18 °C is 360 MPa, which means the material has failed, and the other asphalts are efficient. The failure of CG at −18 °C may be caused by the nitrogen content of biochar. All asphalts fail at −24 °C. Comparing the *S(t)* and *m* values of virgin asphalt and modified asphalt, the low-temperature performance of modified asphalt has a certain degree of deterioration, especially the *S*(*t*) of CG; thus, the deterioration is the most serious. However, it is still much better than SBS-modified asphalt at the same content. The *S*(*t*) and *m* values of CA- and CO-modified asphalt are not much different, and the difference with virgin asphalt is also relatively little, indicating that the deterioration of low-temperature performance is easy to control depending on biochar types.

### 3.3. Asphalt VOC Emission Behavior Analysis

**Figure 14:**
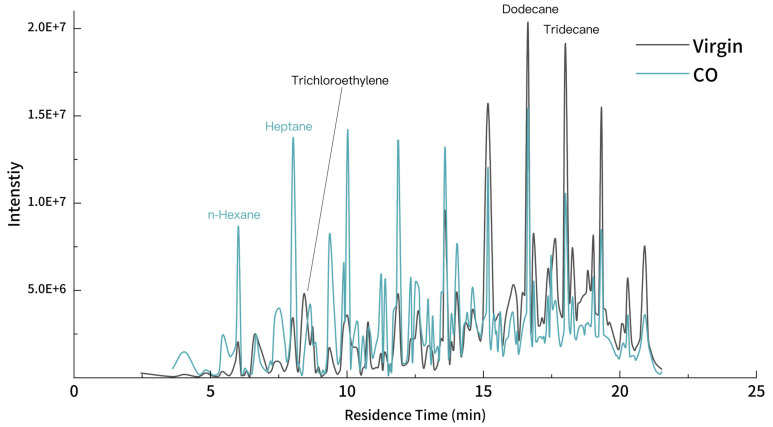
Gas chromatography for modified asphalt.

Based on the results of DSR and BBR tests, CO-modified asphalt demonstrates the best performance among the 3 varieties of modified asphalt. Thus, we evaluated the VOC emission behavior of biochar-modified asphalt. The GC of virgin and CO biochar-modified asphalt is shown in Figure 14. The VOC emission behavior for virgin and modified asphalt are different in the context of residence time. The emission of modified asphalt is more active than virgin asphalt in the first half of the residence time, while virgin asphalt produces more in the second half.

Asphalt VOCs are composed of alkanes, aromatic hydrocarbons, alkenes and a small number of alcohols and aldehydes, of which alkanes and aromatic hydrocarbons such as benzene and toluene account for the majority. The biochar modifier shows an outstanding inhibition on alkanes (n > 10), aldehydes and naphthalene, similar to prior research [22]. Trichloroethylene accounts for the strongest inhibition, reaching 70%, followed by naphthalene, 2,6-dimethyl- with 65%, showing the adsorption to polycyclic aromatic hydrocarbons [60,61,62]. The emissions for alkanes, the component with the highest content in asphalt VOCs, are also effectively controlled [63]. Nearly 60% of the octane, 2,6-dimethyl- emission is inhibited in the asphalt as well as tridecane and pentadecane, at more than 40%. Aldehyde VOCs are also inhibited; 44% of butanal and 34% of pentanal emissions are removed. Though there are increases in the emissions of some VOCs, the entire emissions of asphalt are inhibited by biochar modifier.

## 4. Conclusions

This study prepared biochar-modified asphalt using 3 varieties of biomasses and biochar. Then, we tested the rheological properties at both high and low temperatures for modified asphalt with 6% biochar content. Biochar could significantly enhance the G* and G*/sin δ at mid to high temperature while reducing phase angle δ; therefore, the permanent deformation ability and rutting resistance of modified asphalt are improved. Among the 3 varieties of biochar-modified asphalt, performance differences increase when the temperature is above 50 °C; CG and CO demonstrate better high-temperature performance than CA. Furthermore, the low-temperature deterioration depends greatly on the biochar variety. In the scene of *S(t)*, CO and CG show properties close to virgin asphalt, indicating the controllability of biochar-modified asphalt at the content of 6% by weight of virgin asphalt. The biochar could also influence the VOC emission of modified asphalt. Comprehensively considering high- and low-temperature properties of modified asphalt with biochar properties, it seems like biochar made from biomass with more cellulose is suitable for asphalt modification.

## Figures and Tables

**Table 1:** Conventional properties of virgin asphalt.

Technical Indexes	Units	Results	Requirements	Standards
Penetration (25 °C)	0.1 mm	69	60–80	ASTM D5 [28]
Softening Point	°C	48	≥46	ASTM D36 [29]
Ductility (15 °C)	cm	>100	>100	ASTM D113-17 [30]

**Table 2:** Pore area and volume for biochar.

Variety	CO	CG	CA
Surface area (m^2/^g)	66.4172	11.3879	15.7413
Pore volume (cm^3^/g)	0.0421	0.0137	0.0150
t-Plot micropore area (m^2/^g)	41.1906	9.4560	14.7117
t-Plot micropore volume (cm^3^/g)	0.0176	0.0037	0.0060
BJH mesopore area (m^2/^g)	27.2051	1.0192	1.2887
BJH mesopore volume (cm^3^/g)	0.0256	0.0098	0.0092

**Table 3:** Conventional properties for biochar-modified asphalt.

Sample	Content (%)	Penetration (dmm)			Penetration Index	Softening Point (°C)	Ductility (cm)
		15 °C	25 °C	30 °C			
Virgin	0	29.17	77.62	124.27	−0.32726	49.42	>100
CG	1	28.31	73.30	115.92	−0.14617	49.78	75
	6	23.44	69.82	110.00	−0.78907	51.11	30
	9	18.68	57.23	100.18	−1.25747	51.98	<20
CA	1	25.81	72.11	120.54	−0.71495	50.41	78
	6	23.00	64.27	107.43	−0.71639	51.45	25
	9	22.68	63.97	108.43	−0.80601	52.06	<20
CO	1	28.83	76.64	124.94	−0.39385	50.01	82
	6	24.22	67.87	113.61	−0.73359	50.55	27
	9	23.11	62.69	103.25	−0.52731	51.72	<20

## Data Availability

The original contributions presented in the study are included in the article/Supplementary Materials, further inquiries can be directed to the corresponding author.

## Supplementary Materials

The following supporting information can be downloaded at: https://www.mdpi.com/article/10.3390/ma18071504/s1, Figure S1: Fluorescence microscopic image of biochar modified asphalt; Table S1: Biochar yield in biomass pyrolysis.

## Abbreviations

The following abbreviations are used in this manuscript:

CO	Cotton seed
CA	Camelia seed shell
CG	Coffee ground
DSR	Dynamic shear rheometer
BBR	Bending beam rheometer
VOCs	Volatile organic compounds
GC-MS	Gas chromatography–mass spectrometry
SBS	Styrene–butadiene–styrene block copolymer
SBR	Styrene butadiene rubber
PE	Polyethylene
DSC	Differential scanning calorimetry
TD	Thermal desorption
TTS	Time–temperature superposition
WLF	Williams–Landel–Ferry
