# Supplementary material for: Biochars from Cotton Seed, Camelia Seed Shell, and Coffee Ground in Modification of Asphalt: Fundamental Properties, Rheological Performance, and Inhibition of VOC Emissions
Source: Materials (Basel). 2025 Mar 27;18(7):1504. doi: 10.3390/ma18071504 (PMC11989786; doi:10.3390/ma18071504)
Supplement: Supplementary file 1 [file materials-18-01504-s001.zip › materials-3504303-supplementary.pdf]

Table S1 Biochar yield in biomass pyrolysis

Biomass	CA	CO	CG
Yield Percentage	32%	33%	28%

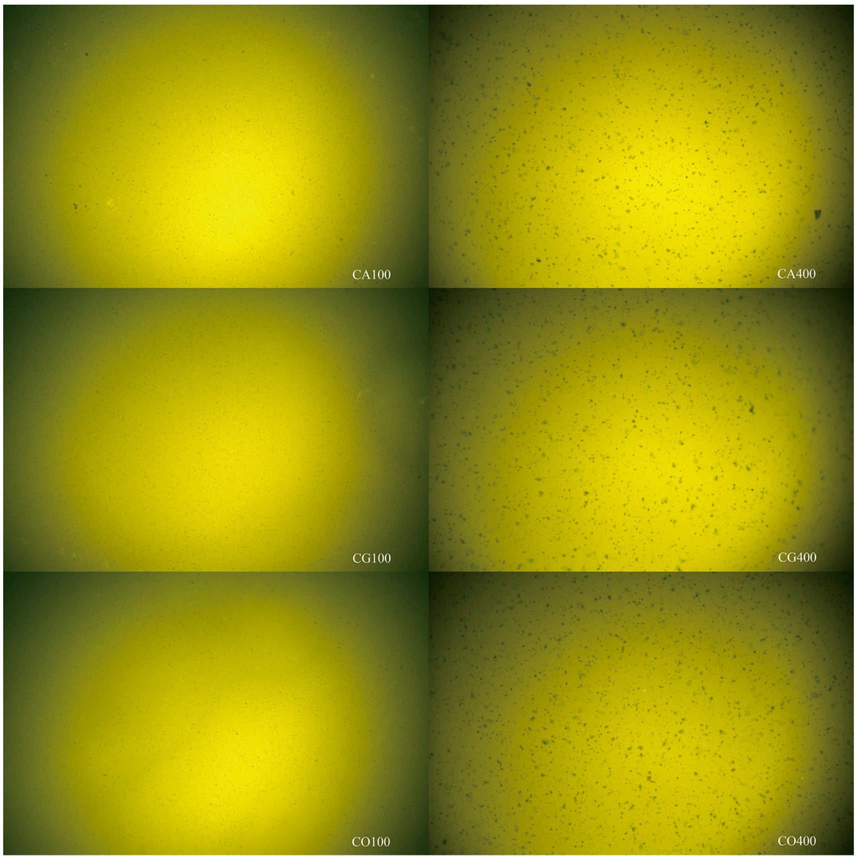

Figure S1 Fluorescence microscopic image of biochar modified asphalt
